# Ticagrelor vs. clopidogrel in dual antiplatelet therapy after coronary artery bypass surgery: a meta-analysis

**DOI:** 10.3389/fcvm.2025.1542437

**Published:** 2025-05-08

**Authors:** Liyuan Wang, Yingying Zhao, Shijie Zhang, Tao Zhang, Jian Song, Yan Yun, Yi Wu, Zhengjun Wang, Xiaochun Ma

**Affiliations:** ^1^ Department of Cardiovascular Surgery, Shandong Provincial Hospital Affiliated to Shandong First Medical University, Jinan, Shandong, China; ^2^ Department of Anesthesiology, Shandong Provincial Hospital Affiliated to Shandong First Medical University, Jinan, Shandong, China; ^3^ Department of Cardiovascular Surgery, Shandong Provincial Hospital, Qilu College of Medicine, Shandong University, Jinan, Shandong, China; ^4^ Department of Radiology, Qilu Hospital of Shandong University, Jinan, Shandong, China

**Keywords:** antiplatelet therapy, coronary artery bypass, clopidogrel, ticagrelor, meta-analyses

## Abstract

**Background:**

Following coronary artery bypass grafting (CABG), the standard treatment regimen typically involves dual antiplatelet therapy (DAPT), which includes a P2Y12 receptor antagonist in combination with aspirin. There is currently no clear consensus regarding the optimal DAPT strategy after CABG. The aim of this meta-analysis was to evaluate and compare the safety and efficacy of ticagrelor vs. clopidogrel in patients post-CABG.

**Methods:**

A meta-analysis of eligible studies of patients undergoing CABG and receiving either aspirin plus clopidogrel (A + C) or aspirin plus ticagrelor (A + T) as antiplatelet therapy, was carried out. The outcomes of interest included all-cause mortality, cardiovascular mortality, major adverse cardiovascular and cerebrovascular events (MACCEs), major bleeding, myocardial infarction, stroke, revascularization, saphenous vein occlusion and total graft occlusion.

**Results:**

4 randomized controlled trials and 3 observational studies (*n* = 2,424) were eligible for final analysis. A + T was associated with a decreased risk of all-cause mortality (OR = 0.47, 95% CI 0.31–0.70, *p* < 0.001, p heterogeneity = 0.80, I^2^ = 0%) and cardiovascular mortality (OR = 0.50, 95% CI 0.31–0.82, *p* = 0.006, p heterogeneity = 0.71, I^2^ = 0%), compared with A + C group. No statistically significant difference was found in the rates of major bleeding (OR = 1.16; 95% CI 0.69–1.96; *p* = 0.57; p heterogeneity = 0.26; I^2^ = 23%) between two groups. Besides, the rates of MACCEs, myocardial infarction, stroke, total graft occlusion, revascularization and saphenous vein occlusion were comparable between two groups (*p* > 0.05).

**Conclusions:**

The meta-analysis presented the evidence supporting the use of A + T post-CAVG in reducing all-cause mortality and cardiovascular mortality, with no increase in bleeding events, in comparison with A + C. Additional RCTs are needed to determine the optimal DAPT after CABG.

## Introduction

Coronary artery bypass grafting (CABG) is the most commonly performed revascularization strategy for treating multivessel and/or left main coronary disease (1, 2). The prognosis of patients after CABG is closely related to platelet aggregation (3, 4). Dual antiplatelet therapy (DAPT), for example, P2Y12 receptor antagonist and aspirin, serves as standard therapeutic regimen after CABG (5). Clopidogrel, an effective antiplatelet agent, has been commonly used worldwide for more than a decade. Clopidogrel is usually metabolized in liver, resulting in a delayed onset of action. Platelet inhibition induced by clopidogrel is highly variable between individuals and more than one-third of patients showing minimal platelet inhibition or “clopidogrel non-response” (6). Ticagrelor is a direct-acting oral P2Y12 receptor antagonist which is reversible and does not require metabolites. Therefore, ticagrelor allows for faster and more potent platelet inhibition compared to clopidogrel (7). According to the PLATO trial, which was conducted in patients with acute coronary syndrome (ACS), ticagrelor has more positive effects in terms of total mortality, cardiovascular events prevention, and stent thrombosis in patients undergoing percutaneous coronary intervention (PCI) compared with clopidogrel (8, 9).

The effectiveness of ticagrelor vs. clopidogrel in patients undergoing CABG surgery has been the subject of several studies but the results were not consistent (10–16). A question remains regarding whether clopidogrel or ticagrelor should be administrated for more favorable outcomes in combination with aspirin following CABG. This meta-analysis study was designed to compare the safety and efficacy outcomes of ticagrelor vs. clopidogrel in patients after CABG.

## Methods

This study's protocol was registered on PROSPERO (CRD42022330721) and reported according to the Preferred Reporting Items for Systematic Reviews and Meta-Analyses guidelines (17). This study was performed under the supervision of the Ethics Committee of Shandong Provincial Hospital affiliated to First Medical University.

## Study selection, inclusion and exclusion criteria

The studies were included investigating adults undergoing CABG and aged 18 or older who received antiplatelet treatment options [aspirin plus clopidogrel (A + C) or aspirin plus ticagrelor (A + T)], and reported postoperative mortality, cardiocerebrovascular and/or bleeding outcomes for each treatment arm.

Inclusion criteria:

(A) published randomized controlled trials (RCTs) or observational studies as full-text, peer-reviewed articles;
(B) recruiting adult patients undergoing CABG;
(C) patients received DAPT with either A + T or A + C due to the specific treatment plan;
(D) interested postoperative outcomes included in the studies: all-cause mortality, cardiovascular mortality, major adverse cardiovascular and cerebrovascular events (MACCEs), major bleeding, myocardial infarction, stroke, revascularization, saphenous vein occlusion and total graft occlusion;
(E) study-level data available for statistical analysis.

Exclusion criteria:

(a) case reports, editorials, reviews, and meta-analysis articles and non-English studies;
(b) studies unpublished, published in duplicate, or with insufficient data;
(c) studies or subgroups of patients who were preoperatively exposed to other anticoagulants (such as heparin and warfarin).

If there was any overlap in the patient populations across multiple studies conducted at the same center, we opted to include only the study with the longest follow-up period or the largest patient cohort.

### Search strategy and data extraction

We conducted a thorough search of Embase, Cochrane, and Web of Science databases from 2000 to 2024 to identify relevant studies. A comprehensive search strategy was developed to identify articles comparing the outcomes of A + C vs. A + T after CABG. The search terms used either alone or in combination included CABG, coronary artery bypass graft, coronary artery bypass surgery, aspirin, clopidogrel, ticagrelor, dual antiplatelet therapy, DAPT and etc.

Abstracts identified through our search strategies were independently reviewed by two authors (Xiaochun Ma and Liyuan Wang). Full-text articles that potentially met the criteria were then reviewed by another two authors (Zhengjun Wang and Yingying Zhao) to decide the inclusion in the analysis. Additionally, the reference lists of retrieved articles were manually checked to include any potential publications. Disagreements were resolved through further discussion among all authors.

Data extraction was independently conducted by two authors (Xiaochun Ma and Zhengjun Wang). Extracted data included details the study design and quality, participant demographics, baseline characteristics, and the outcomes of interest. The efficacy outcomes were mortality (all-cause and cardiovascular), myocardial infarction, stroke, major adverse cardiovascular and cerebrovascular events (MACCEs), revascularization, saphenous vein occlusion and total graft occlusion and safety outcome was major bleeding. MACCE is a composite endpoint commonly used in cardiovascular research to evaluate the overall impact of a treatment or intervention. It typically includes a combination of the following critical clinical outcomes: cardiovascular mortality, myocardial infarction(MI), stroke, revascularization and hospitalization for unstable angina or heart failure. Similarly, disagreements were resolved by a further discussion among all authors.

### Quality assessment

The quality assessment independently was conducted by two authors (Yan Yun and Liyuan Wang). The Newcastle-Ottawa Quality Assessment Scale (NOS) was used to assess the risk of bias of all observational studies (http://www.ohri.ca/programs/clinical_epidemiology/oxford.asp.). In articles reporting randomized controlled trials (RCTs), the risk of bias was assessed using the RoB2 (Revised Cochrane risk of bias tool for randomized trials) (18).

### Statistical analysis

Data were expressed as mean ± SD for continuous variables and frequency with percentage for categorical variables. Pooled analyses were performed using the Mantel-Haenszel random-effects model to calculate the odds ratio (OR) with a 95% confidence interval (CI) for all outcomes reported by at least two studies. A 2-sided *P* value of less than 0.05 was considered statistically significant. We used the chi-square test of heterogeneity with statistical significance set at a 10% level (*p* = 0.10) to test for heterogeneity. Additionally, we utilized the I^2^ statistic to estimate the inconsistencies between included studies. Publication bias was assessed using Funnel plots and Egger's statistics, with a *P* value of less than 0.05 considered as significant. Reviewer Manager V.5.4 and Stata 14.0 were applied for the pooled analyses.

## Results

### Eligible studies

**Figure 1:**
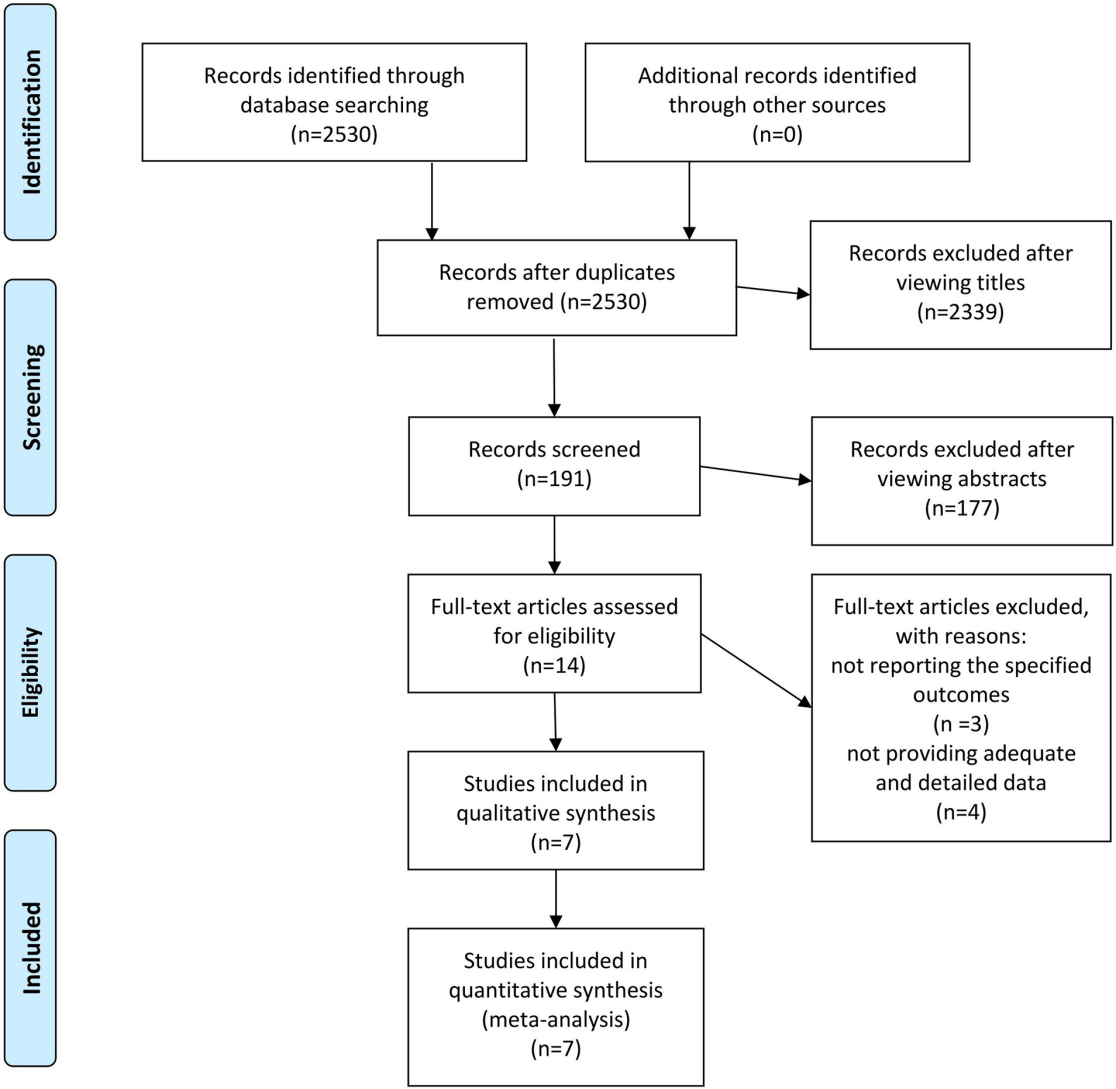
The PRISMA recommended flow-diagram depicting the methodology of article selection for this meta-analysis.

A total of 2,530 potentially eligible studies were initially identified through the literature search. Upon reviewing the titles, 2,339 studies were excluded, leaving 191 studies that were considered potentially eligible. Subsequent abstract review resulted in the exclusion of an additional 177 studies. Ultimately, 14 studies [comprising 8 randomized controlled trials (RCTs) and 6 observational studies] were selected for full-text assessment to determine their inclusion. Then a collaborative review of the full-text articles by all authors resulted in the exclusion of 3 studies for not reporting the specified endpoints and 4 studies for not providing adequate and detailed data (Figure 1).

### Study characteristics

**Figure 2:**
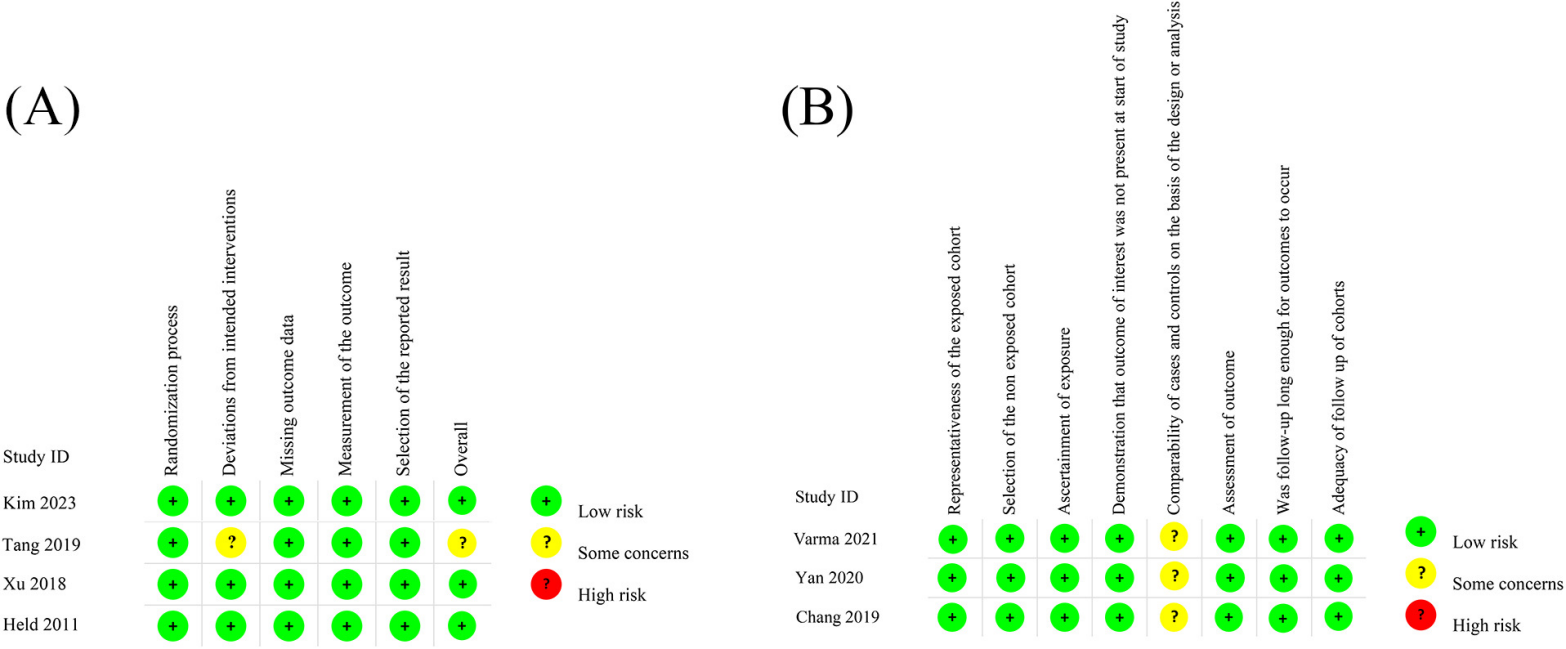
The assessment of RCTs using the Cochrane Risk of Bias Tool **(A)** the assessment of observational studies using NOS **(B)**.

A total of 4 randomized controlled trials (RCTs) and 3 observational studies encompassing 2,424 patients met our criteria and were included in our meta-analytical review. The characteristics of the included studies were summarized in Table 1. Following the assessment using the Cochrane Risk of Bias Tool, three RCTs were classified as low-risk while the remaining one as some concern (Figure 2A; Supplementary Table 1). Employing the Newcastle-Ottawa Scale, all observational studies were regarded as high quality (Figure 2B; Supplementary Table 2).

**Table 1:** Outline of studies included in the meta-analysis.

Characteristics	Kim 2023	Tang 2021	Xu 2018	Held 2011	Varma 2021	Yan 2020	Chang 2019
Year	2023	2021	2018	2011	2021	2020	2019
Country	Korea	China	China	Sweden	India	China	China
Study type	RCT	RCT	RCT	RCT	Retrospective study	Retrospective study	Retrospective study
Sample size	204	147	137	1,258	288	121	269
Design	AT:102	AT:70	AT:70	AT:629	AT:144	AT:54	AT:169
	AC:102	AC:77	AC:67	AC:629	AC:144	AC:67	AC:100
Follow-up, years	4.3 (2.9-5.2)	1	0.1	Unknown	1	2.5 (1.9–2.9)	1.8
Mean age	67.9	64.4	59.5	64	60.7	60	64
Male, %	78.9	82.3	75	78.9	82.6	77.7	74.7
BMI, kg/m^2^	AT:24.8	AT:25.4	Unknown	AT:27.4	Unknown	AT:25.5	Unknown
	AC:23.5	AC:25.5		AC:26.9		AC:25.7	
Diabetes mellitus, %	AT:38.2	AT:32.9	AT:67.1	AT:30.5	AT:58.3	AT:38.9	AT:51
	AC:40.2	AC:32.5	AC:67.1	AC:32.9	AC:58.3	AC:44.8	AC:52
Hypertension, %	AT:47.1	AT:60	AT:68.6	AT:68.5	AT:66.7	AT:64.8	AT:53
	AC:43.1	AC:63.6	AC:62.6	AC:67.1	AC:68.1	AC:67.2	AC:66
LVEF, %	AT:48.5	AT:59.5	AT:58.4	Unknown	Unknown	AT:60	AT:50
	AC:48.6	AC:61.8	AC:59.4			AC:62	AC:52
Previous MI, %	AT:3.9	AT:30.0	AT:35.7	AT:19.6	AT:41.7	AT:29.6	AT:8
	AC:3.9	AC:35.1	AC:40.0	AC:20.8	AC:38.9	AC:47.8	AC:13
Outcomes	①②③④⑤⑥⑦⑨	①②③④⑤⑥⑧⑨	①②③④⑤⑦⑧⑨	①②③④⑤⑨	①③④⑤⑨	①③④⑤⑦⑨	①③④⑤⑦⑨

① All-cause mortality; ② Cardiovascular mortality; ③ MACCEs; ④ Myocardial infarction; ⑤ Stroke; ⑥ Total graft occlusion; ⑦ Revascularization; ⑧ Saphenous vein occlusion; ⑨ Major bleeding.

RCT, randomized controlled trial; MI, myocardial infaction; BMI, body mass index.

### Efficacy outcomes

All-cause mortality, cardiovascular mortality, MACCEs and myocardial infarction.

**Figure 3:**
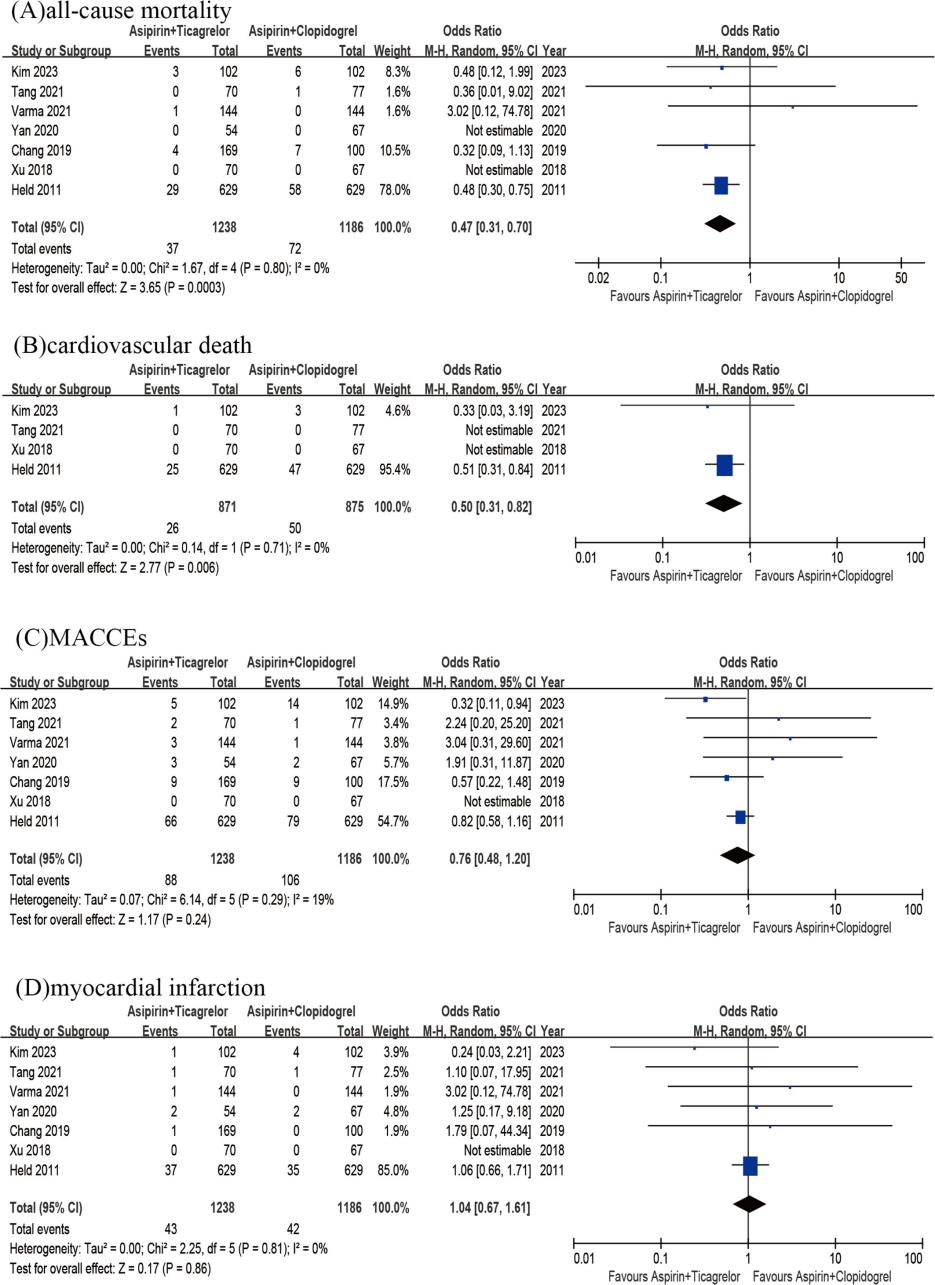
All-cause mortality of A + T group versus A + C group **(A)**; cardiovascular death od A + T group versus A + C group **(B)**; MACCEs of A + T group versus A + C group **(C)**; myocardial infarction of A + T group versus A + C group **(D)**.

Pooling the results of 4 RCTs and 3 observational studies (*n* = 2,424) together showed a significant difference in the rate of all-cause death between the 2 groups. The A + T group exhibited a significantly lower rate of all-cause death compared to A + C group (OR = 0.47, 95% CI 0.31–0.70, *p* < 0.001, p heterogeneity = 0.80, I^2^ = 0%) (Figure 3A). Then the A + T group exhibited a significantly lower rate of cardiovascular mortality (OR = 0.50, 95% CI 0.31–0.82, *p* = 0.006, p heterogeneity = 0.71, I^2^ = 0%) in comparison with the A + C group, showed by a pooled analysis of 4 studies (*n* = 1,746) (Figure 3B). A pooled result of 4 RCTs and 3 observational studies (*n* = 2,424) did not demonstrate any significant difference in the rates of MACCEs between two groups (OR = 0.76; 95% CI 0.48–1.20; *p* = 0.24; p heterogeneity = 0.29; I^2^ = 19%) (Figure 3C). Besides, analyzing the results of 4 RCTs and 3 observational studies (*n* = 2,424) showed no significant difference in the rates of MI between two groups (OR = 1.04; 95% CI 0.67–1.61; *P* = 0.86; P heterogeneity = 0.81; I^2^ = 0%) (Figure 3D).

Stroke, total graft occlusion, revascularization and saphenous vein occlusion.

**Figure 4:**
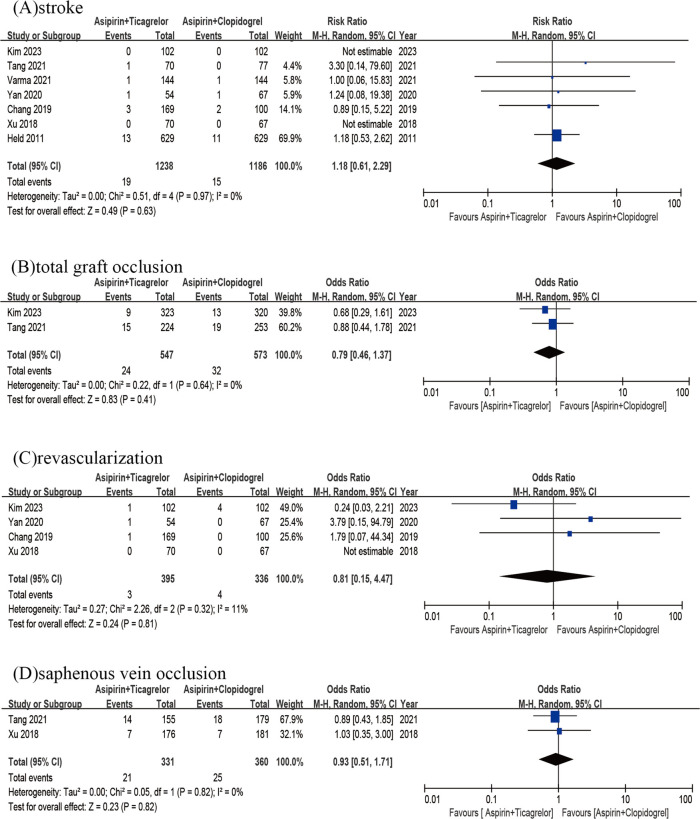
Stroke of A + T group versus A + C group **(A)**; total graft occlusion of A + T group versus A + C group **(B)**; revascularization of A + T group versus A + C group **(C)**; saphenous vein occlusion of A + T group versus A + C group **(D)**.

The findings from 7 studies did not reveal any significant variation in the rates of stroke (OR = 1.18; 95% CI 0.61–2.29; *p* = 0.63; p heterogeneity = 0.97; I^2^ = 0%) (Figure 4A). Then the incidence of total graft occlusion was similar between two groups (OR = 0.79; 95% CI 0.46–1.37; *p* = 0.41; p heterogeneity = 0.64; I^2^ = 0%) (Figure 4B). A pooled analysis of 4 studies (*n* = 931) revealed that the rate of revascularization did not decrease with the use of the A + T compared to A + C (OR = 0.81; 95%CI 0.15–4.47; *p* = 0.81; p heterogeneity = 0.32; I^2^ = 11%) (Figure 4C). Additionally, the results of 2 studies (*n* = 691) alone did not demonstrate any significant difference in the rates of saphenous vein occlusion (OR = 0.93; 95%CI 0.51–1.71; *p* = 0.82; p heterogeneity = 0.82; I^2^ = 0%) (Figure 4D).

### Safety outcomes

#### Major bleeding

**Figure 5:**
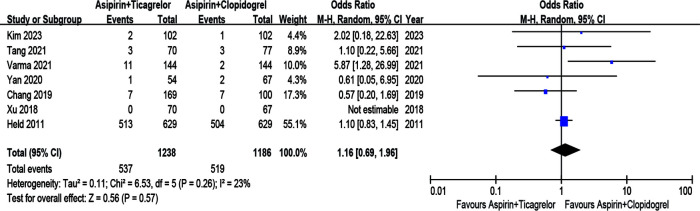
Major bleeding of A + T group versus A + C group.

The risk of major bleeding between the A + T group and A + C group was comparable, showed by pooling the results of 4 RCTs and 3 observational studies (*n* = 2,424) (OR = 1.16; 95% CI 0.69–1.96; *p* = 0.57; p heterogeneity = 0.26; I^2^ = 23%) (Figure 5).

### Publication bias

Publication bias was evaluated through the use of the Funnel plots and Egger's statistics (p > 0.05), revealing no obvious publication bias for any of the outcomes.

## Discussion

For decades CABG has been proven to be an effective surgical intervention and to reduce significantly morbidity and mortality, especially in patients with multivessel disease and left main coronary artery disease (19, 20). Although its efficacy has been confirmed, there is still controversy over the optimal postoperative antiplatelet strategy following CABG. Despite aspirin being the mainstay of treatment, patients still face up to a high residual cardiovascular risk after CABG due to platelet activation and thrombus formation as well as heavy atherosclerotic burden. Ticagrelor and clopidogrel are commonly used antiplatelet agents in this setting, but their comparative efficacy and safety profile after CABG surgery remain a topic of debate. Previously several studies have compared the efficacy and safety outcomes of ticagrelor and clopidogrel in patients undergoing CABG surgery but the findings from these studies were inconsistent (10–16). Recently, it was found that major adverse cardiovascular events (MACEs) were similar between DAPT with ticagrelor and DAPT with clopidogrel following CABG (13, 14). In contrast, a meta-analysis indicated that ticagrelor-based regimens might reduce mortality and MACEs without an increased risk of bleeding when compared to aspirin monotherapy or aspirin combined with clopidogrel in patients undergoing CABG (21).

The purpose of this meta-analysis was to combine the results of these studies to provide a more comprehensive and reliable summary of the existing evidence that might not be apparent in individual studies alone. In this meta-analysis we have compared the safety and efficacy endpoints of aspirin plus ticagrelor (A + T) vs. aspirin plus clopidogrel (A + C) in patients after CABG from 7 studies and found an advantage of postoperative administration of ticagrelor in preventing all-cause mortality and cardiovascular mortality compared to clopidogrel with a similar risk of major bleeding between two groups.

Unlike clopidogrel, which relies on liver enzyme activation, ticagrelor is known for its quick onset of action and potent inhibition of platelet aggregation, leading to clear pharmacokinetic benefits over clopidogrel. The PLATO trial demonstrated that ticagrelor was associated with a lower rate of cardiovascular death, myocardial infarction, and stroke compared to clopidogrel in patients with acute coronary syndrome (8, 9). Besides, a minority of patients exhibit polymorphism within the CYP2C19 gene, resulting in resistance to clopidogrel but generally not affecting the efficacy of ticagrelor or prasugrel (22, 23). Consequently, these individuals face a higher risk of vascular events if treated with clopidogrel. Several studies have demonstrated that within the first year following CABG, ticagrelor outperforms clopidogrel in preserving saphenous vein graft (SVG) patency and reducing MACEs (24, 25). In our study we observed a significant association between A + T and lower rates of all-cause and cardiovascular mortality compared with A + C in patients undergoing CABG. However, the underlying mechanism remains unclear, as there were no significant differences in other vascular endpoints such as myocardial infarction, stroke, MACCEs, graft occlusion and revascularization.

While ticagrelor has shown promising efficacy outcomes, its safety profile must also be considered. The PLATO trial reported a higher rate of non-CABG-related major bleeding events with ticagrelor compared to clopidogrel. And ticagrelor was linked to a higher incidence of major bleeding, including fatal intracranial bleeding and other types of bleeding not related to CABG (24). Additionally, Jing et al. reported that ticagrelor raised the risk of bleeding events in Asian patients (26). However, reversible binding of ticagrelor to the P2Y12 receptor might provide an advantage in patients requiring urgent surgery. On the other hand, clopidogrel has a longer duration of action and a lower risk of bleeding complications, making it a preferred option in some patients. It is noteworthy in this study that there were no significant differences in major bleeding events observed between the A + C and A + T groups during the post-CABG follow-up period.

Mortality is a robust and patient-centered endpoint, reflecting the ultimate impact of a treatment on survival. The observed mortality benefit with ticagrelor may be attributed to its more potent and consistent platelet inhibition compared to clopidogrel, which could be particularly advantageous in high-risk post-CABG patients. However, we acknowledge that the lack of significant differences in other endpoints raises important questions. This discrepancy could stem from several factors. Firstly, the relatively low event rates for myocardial infarction, stroke, and MACCEs in the included studies may limit the ability to detect significant differences. Secondly, variations in study populations, follow-up durations, and definitions of endpoints across studies may contribute to inconsistent findings. Thirdly, the mechanisms driving mortality reductions (e.g., prevention of fatal or non-cardiac deaths) may differ from those influencing other endpoints.

For AC (anti-coagulation) therapy, in Xu's study, the exclusion criterion was AC therapy that could not be withheld. However, it is unclear whether some patients received AC therapy during the observation period. Unfortunately, detailed data on AC therapy use were not reported in the study, limiting our ability to account for its potential impact on bleeding outcomes. Besides, in Kim's study, therapy was switched if clopidogrel resistance was detected. This might introduce variability in the treatment groups, as patients with clopidogrel resistance may have different outcomes compared to those without. While we included Kim's study in our analysis due to its relevance, we recognize that this factor could affect the comparability of the results. This issue highlights the need for standardized protocols in future studies to minimize such variability.

Our results suggested that ticagrelor might be preferred over clopidogrel following CABG procedures. The existing guidelines mentioned the potential use of clopidogrel or ticagrelor, and in the European guidelines, prasugrel is also considered as an option for the P2Y12 inhibitor agent in specific patients undergoing CABG. Therefore, our findings provide support for considering ticagrelor as the preferred choice in this particular scenario.

### Limitations

Firstly, we pooled the data from both RCTs and observational studies in the analysis. 3 observational studies were included which had inherent biases from study design. We acknowledge the potential for bias introduced by combining these study types, as observational studies are more susceptible to confounding factors and limitations in internal validity compared to RCTs. While RCTs are considered the gold standard for evaluating treatment efficacy due to their rigorous design and minimization of bias, observational studies provide valuable real-world evidence that complements RCT findings. In the context of our topic on DAPT after CABG, RCTs are often limited in number and may not fully capture the heterogeneity of clinical practice or long-term outcomes. Observational studies, despite their inherent limitations, offer insights into broader patient populations and real-world effectiveness, which are critical for informing clinical decision-making. To address potential bias, we rigorously assessed study quality using the Cochrane Risk of Bias Tool for RCTs and the Newcastle-Ottawa Scale for observational studies. We also emphasized the need for further high-quality RCTs to confirm our findings.

Secondly, some results had a small number of included studies and events, leading to insufficient analytical power. Therefore, these findings require larger randomized trials for validating the conclusions.

Thirdly, the limited number of studies and patients made it difficult for further subgroup analysis, such as providing different treatment recommendations for the acute myocardial infarction, unstable coronary artery disease and stable coronary artery disease subgroups. These factors could influence the effectiveness and safety of antiplatelet regimens and provide more insights into the clinical applicability of the results. However, due to the limited number of studies included in our meta-analysis, particularly for specific subgroup analyses, conducting meaningful subgroup analyses was not feasible. The small sample size within potential subgroups would have significantly reduced the statistical power and reliability of the findings, potentially leading to misleading conclusions. We have explicitly acknowledged this limitation in the revised manuscript and emphasized the need for further research to explore these important factors in larger, more diverse populations.

Fourthly, there was heterogeneity in the demographic characteristics of patients, treatment and follow-up duration as well as endpoint reported in the studies.

Fifth, all studies did not report outcomes of different antiplatelet regimens under different surgical strategies, such as total arterial CABG vs. mixed arterial and venous CABG and off-pump vs. on-pump CABG.

Sixth, the interpretation of funnel plots and Egger's tests should be approached with caution due to the limited number of studies, particularly for specific outcomes. The potential for publication bias cannot be entirely ruled out, even in the absence of significant statistical evidence. We have reinforced the need for additional high-quality studies, particularly RCTs, to validate our findings and provide more robust evidence on the comparative effectiveness of ticagrelor vs. clopidogrel in DAPT after CABG.

Seventh, our analysis did not account for potential ethnic/racial differences in outcomes due to insufficient data. The majority of included studies were conducted in Asian populations, with one Swedish cohort, yet none provided stratified racial/ethnic subgroup analyses. Heterogeneity in racial documentation standards across studies and the absence of ethnic data precluded meaningful exploration of this factor.

## Conclusions

In conclusion, the meta-analysis provided the evidence in favor of using aspirin plus ticagrelor (A + T) post-CABG to reduce all-cause mortality and cardiovascular mortality without an increase in bleeding events compared with aspirin plus clopidogrel (A + C). Further randomized controlled trials (RCTs) are required to confirm the most effective dual antiplatelet regimens following CABG.

## Data Availability

The original contributions presented in the study are included in the article/Supplementary Material, further inquiries can be directed to the corresponding authors.
