# Supplementary figures and images for: Ticagrelor vs. clopidogrel in dual antiplatelet therapy after coronary artery bypass surgery: a meta-analysis
Source: Front Cardiovasc Med. 2025 May 8;12:1542437. doi: 10.3389/fcvm.2025.1542437 (PMC12095165; doi:10.3389/fcvm.2025.1542437)

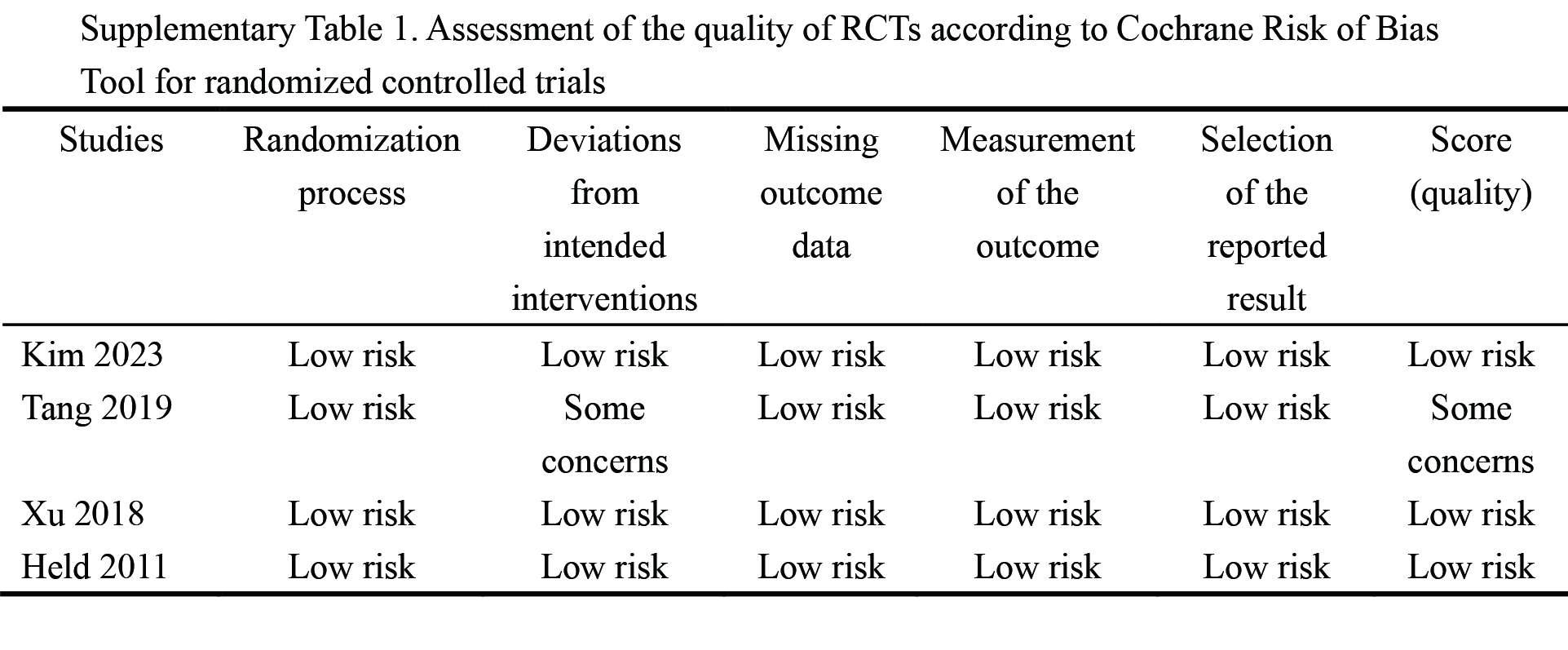

Supplement: Supplementary file 1 [file Image1.tif]

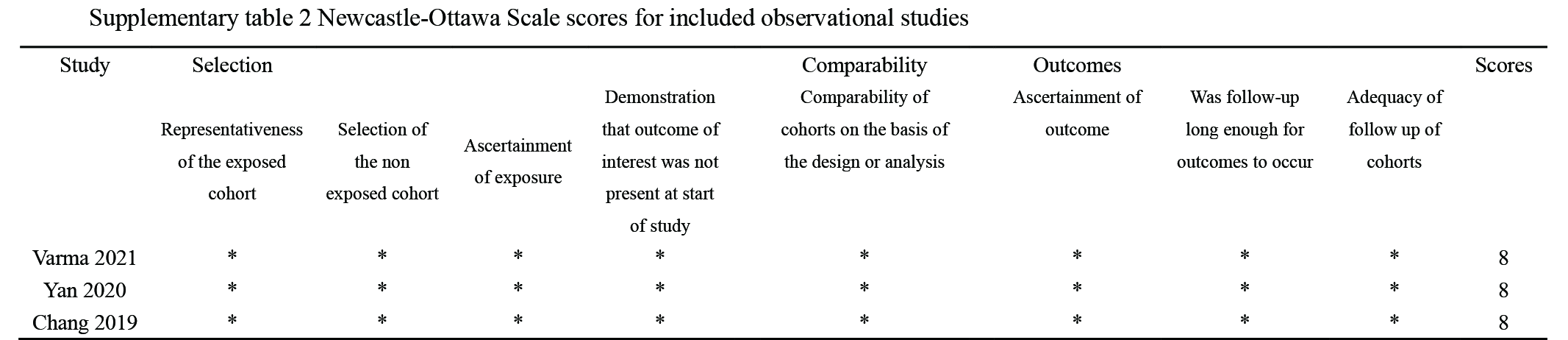

Supplement: Supplementary file 2 [file Image2.tif]
